# Toward a Conversational Agent to Support the Self-Management of Adults and Young Adults With Sickle Cell Disease: Usability and Usefulness Study

**DOI:** 10.3389/fdgth.2021.600333

**Published:** 2021-01-29

**Authors:** David-Zacharie Issom, Marie-Dominique Hardy-Dessources, Marc Romana, Gunnar Hartvigsen, Christian Lovis

**Affiliations:** ^1^Department of Radiology and Medical Informatics, Faculty of Medicine, University of Geneva, Geneva, Switzerland; ^2^INSERM U1134 Biologie Intégrée du Globule Rouge, Paris, France; ^3^Department of Computer Science, UiT the Arctic University of Norway, Tromsø, Norway

**Keywords:** sickle cell disease, conversational agent, mhealth, high-fidelity prototype, user testing and evaluation, usability evaluation, self-management, patient innovation

## Abstract

Sickle cell disease (SCD) is the most common genetic blood disorder in the world and affects millions of people. With aging, patients encounter an increasing number of comorbidities that can be acute, chronic, and potentially lethal (e.g., pain, multiple organ damages, lung disease). Comprehensive and preventive care for adults with SCD faces disparities (e.g., shortage of well-trained providers). Consequently, many patients do not receive adequate treatment, as outlined by evidence-based guidelines, and suffer from mistrust, stigmatization or neglect. Thus, adult patients often avoid necessary care, seek treatment only as a last resort, and rely on self-management to maintain control over the course of the disease. Hopefully, self-management positively impacts health outcomes. However, few patients possess the required skills (e.g., disease-specific knowledge, self-efficacy), and many lack motivation for effective self-care. Health coaching has emerged as a new approach to enhance patients' self-management and support health behavior changes. Recent studies have demonstrated that conversational agents (chatbots) could effectively support chronic patients' self-management needs, improve self-efficacy, encourage behavior changes, and reduce disease-severity. To date, the use of chatbots to support SCD self-management remains largely under-researched. Consequently, we developed a high-fidelity prototype of a fully automated health coaching chatbot, following patient-important requirements and preferences collected during our previous work. We recruited a small convenience sample of adults with SCD to examine the usability and perceived usefulness of the system. Participants completed a post-test survey using the System Usability Scale and the Usefulness Scale for Patient Information Material questionnaire. Thirty-three patients participated. The majority (64%) was affected by the most clinically severe SCD genotypes (Hb SS, HbSβ0). Most participants (94%) rated the chatbots as easy and fun to use, while 88% perceived it as useful support for patient empowerment. In the qualitative phase, 72% of participants expressed their enthusiasm using the chatbot, and 82% emphasized its ability to improve their knowledge about self-management. Findings suggest that chatbots could be used to promote the acquisition of recommended health behaviors and self-care practices related to the prevention of the main symptoms of SCD. Further work is needed to refine the system, and to assess clinical validity.

## Introduction

Sickle cell disease (SCD) is the most prevalent monogenic disorder worldwide, and represents an increasing global health issue (1). Annually, over 300'000 infants are born with it and this number is expected to raise above 400'000 in 30 years (2). SCD is mainly widespread throughout Africa, India, Middle East, the Caribbean and Mediterranean countries. With population movements, the distribution of SCD has spread worldwide, both in high-income and lower-income countries. In Africa, 50%-90% of affected children die before their fifth birthday, and up to 90% of survivors will not reach 18 years of age (3). In contrast, in high-income countries, where access to high quality healthcare is more available, median life expectancy is estimated at 50 years (4).

SCD is an autosomal recessive blood disorder caused by a change in the beta globin gene (2). People with SCD may have any of a number of hemoglobin genotypes of variable clinical severity (e.g., HbSS, HbSC, HbSβ0, HbSE, Hb SLepore, HbSD) (5). Furthermore, SCD presents a complex pathophysiology, shows considerable clinical variability and is sensitive to various modifying factors (e.g., socio-economical, genetic, biochemical, environmental and behavioral) (2, 6). The disease causes severe complications such as chronic hemolytic anemia and vaso-occlusive pain crises (VOCs) (7). Patients often suffer from a wide spectrum of acute symptoms and chronic complications such as organ damages, chronic inflammation, lung diseases, or sleep disturbances. With aging, SCD manifestations worsen, requiring comprehensive, preventative life-long care.

Simple public health measures and prevention of acute complications include: newborn screening, parental and patient education, antibiotic prophylaxis, up-to-date immunizations, and routine health management with a hematologist or a healthcare provider with expertise in SCD (5). The last decade has seen important advances in the treatment of SCD (8). However, treatments remain limited to a few approved disease-modifying therapies (i.e., Hydroxyurea, Voxelotor, l-Glutamine, Crizanlizumab), symptomatic pain medication, and chronic blood transfusions. Additionally, impacted by their social determinants of health (e.g., genetics, socio-economic status, access to quality healthcare, environmental factors), some patients respond poorly to treatments (9). Consequently, they will have little reduction of symptoms, and the disease will continue to progress. Finally, the only established cure, hematopoietic stem cell transplant is not available to most patients, because of the need for compatible donors, potentially life-threating side effects, procedure-related toxicities, and high costs (10).

Furthermore, compared to the pediatric population, access to comprehensive and preventative care is challenging for the adult and young adult (AYA) population (11). These disparities are mainly caused by shortages of well-trained healthcare providers, and under-resourced specialized sickle cell centers. With such health inequity, mistrust levels are high among AYA with SCD, and many miss routine care appointments (12). Thus, AYA with SCD rely on sub-optimal emergency care (13). Because they often are prone to stigma, neglect or under-treatment, patients often avoid emergency departments and seek treatment only as a last resort, which negatively impacts long-term health outcomes and may lead to early mortality (14).

Consequently, to avoid disastrous consequences and maintain a certain level of Quality of Life, patients must take charge of their own health. Self-management in SCD is key to reduce symptoms frequency and extend quality-adjusted life expectancy (4, 15, 16). However, similarly to other chronic diseases requiring complex healthcare needs (17, 18), SCD self-management is particularly demanding (19). Patients must pay attention to numerous precipitating factors of symptoms, including inadequate eating behaviors, stress, hypoxia, acidosis, infections, dehydration, fatigue, physical exertion, climate (e.g., extreme of temperatures, wind), air pollution, altitude (10, 20, 21). As well, SCD self-management aspects cover self-care in hospitalization, post-hospitalization care, hospital-at-home care, preventive care, health maintenance, self-monitoring, self-diagnosis, or self-treatment (22, 23). Managing effectively such complexity requires an array of skills (e.g., high cognitive capabilities, good disease-specific knowledge, high levels of self-efficacy, problem solving) that only few patients possess (16, 18, 24, 25).

Given the limited resources of healthcare service delivery, scalable and low-cost Mobile health (mHealth) interventions could offer a potential route to support the self-management needs of this population (26). To date, although evidence on feasibility and acceptability is robust, mHealth interventions targeting AYA with SCD are rare, and only offer limited features (27–32). Most research-based apps for SCD typically focus on pain symptoms monitoring or medication adherence. The few publicly available self-management apps propose manual self-tracking (e.g., pain symptoms, hydration) (33, 34). All these features are typically perceived by patients as an additional burden (35). Therefore, long-term engagement in these apps is low, and frequency of use decreases over time. With discontinued or inconsistent use, it is less likely that the intended effectiveness of the mHealth interventions can be realized. Consequently, patients who stand to benefit most from them are least likely to download or use them (36–38).

To address these gaps, prior studies have demonstrated that long-term engagement can be increased when mHealth interventions provide clear utility, personalization, ease of use and seamless integration in daily life (39–43). In addition, recent studies have demonstrated that using conversational agents (i.e., software that imitate communication with humans) such as chatbots could effectively encourage behavior changes and improve health outcomes (44, 45). Chatbots have several natural advantages: anonymity, asynchronicity, personalization, scalability (46). In addition, by living inside messaging apps, increasingly the most used feature of smartphone users (47), conversational agents provide a convenient way to engage with users where they already are. Consequently, this could lead to high acceptance, and ease long-term user engagement through the development of an attachment bond between the user and the system (48, 49). Finally, creating conversational agents that are empathetic and effective could reduce the need for in-person appointments and direct patient-provider interaction, providing much-needed scalability to relieve pressure on the current limited health care resources.

To our knowledge, no work has been done to design chatbots for the specific self-management needs of people with SCD. There is a clear need to understand the usefulness of conversational agents to achieve this intended outcome, and facilitate the user experience with these particular agents. This information can then be used to determine the direction that these technologies are most likely to follow. Consequently, we developed a high-fidelity prototype of a fully automated mHealth coaching app (TREVOR, the sickle cell robot coach), following patient-important requirements and preferences collected during our previous work (50–52).

TREVOR has been designed to deliver simple text-based messages and media objects (e.g., videos, podcasts) to patients in an empathetic way. The chatbot has three objectives. The first aim is to educate patients with evidence-based knowledge on SCD self-management. The second is to inform them on the self-care practices that other patients have ranked as effective in reducing the incidence of VOCs. The third is to connect patients together for community peer-support.

This paper is the last component of a study from which preliminary results have already been published (53). This prior publication was the first to elaborate on mHealth coaching apps to promote the knowledge acquisition of recommended health behaviors related to the prevention of SCD main symptoms. The authors explored a smaller subset of the studied population and focused on a partial analysis of patients' perceived usefulness of the information provided by the chatbot.

The objectives of this paper are to (1) present the chatbot, (2) assess its usability using a group testing approach, (3) evaluate patients' perceived usefulness of the information provided by the system, and to (4) evaluate patient satisfaction in the provided features.

## Materials and Methods

### Study Design

The study was divided in two phases. Phase 1 employed a mixed-methods design, combining quantitative and qualitative data to explore patients' experiences of using the chatbot. Phase 2 exploited qualitative design to explore patients' satisfaction and specific recommendations for better designing conversational agents.

### Participants

To be eligible, AYA had to be diagnosed with SCD, possess a smartphone with Facebook Messenger pre-installed, be at least 16 years old [minimum age to have a Facebook account in Europe (54)], and be able to understand French. Individuals who had been cured through bone marrow transplantation gene therapy were excluded. AYA with known cognitive impairment or disabilities that would interfere with completion were accompanied by their caregiver.

Patients were recruited in June 2019 through patient associations and healthcare providers in Guadeloupe and Martinique, prior to the World Sickle Cell Awareness Day. Recruitment was carried out via ads posted on social networks (i.e., WhatsApp, Facebook), as well as through paper posters disseminated in three hospitals (2 in Guadeloupe, 1 in Martinique), and through contacts with sickle cell associations present locally. Interested individuals were prompted to contact the research coordinators from the CAREST network, and could then register for participation. Prior to beginning the evaluation, individuals were invited to download Facebook Messenger via the project webpage or directly via Apple App Store or Google Play Store.

### Procedure

All participants gave informed consent before the evaluation and all responses were anonymous. Tests were executed by the first author (DZI), an expert patient, two medical students, two specialized nurses and one medical doctor. Patients were invited to a room in small groups so facilitators could make sure to observe one user at a time. All the tests were video-recorded. Evaluations were conducted using the guide presented in Table 1. The guide was developed so patients would have to perform six tasks in the chatbot following a scenario. These tasks were used to simulate the actions that a user may be required to perform by interacting with the chatbot. The goal was to see if users could find information quickly, navigate the application easily, and thus allow us to identify strengths and weaknesses in the system.

**Table 1:** Tasks that patients had to perform.

**#**	**Task (duration)**
1	Provide basic information, demographics and general health status (10 mn)
2	Enter your own self-care practices (10mn)
3	See the best practices of the patient community (5mn)
4	See your evaluation (5mn)
5	Request a health coaching from a human (1mn)
6	Talk to a coach or join the online peer-support community (1mn)

First, AYA received a presentation of the mHealth coaching chatbot to understand its aim. Anytime, participants could ask questions relevant to the research aims. Second, participants completed baseline demographic measures. Third, patients tested the chatbot during about 45 min. Fourth, participants completed a post-test survey to measure perceptions of usability and usefulness, which lasted about 15 min. Finally, AYA could share additional insights on their satisfaction using the chatbot by completing a 7 items non-mandatory questionnaire. See Table 2.

**Table 2:** Debriefing questionnaire.

**#**	**Task (duration)**
Q1	What did you think of the chatbot, as a whole?
Q2	Did you have difficulty finding the information? If so why?
Q3	What are the strengths of this chatbot?
Q4	In your opinion, are there points to improve, if so which ones?
Q5	If TREVOR was omniscient, what would you like to ask him?
Q6	If you don't already have it, would you install Messenger (or another messaging app) for TREVOR? Why?
Q7	Do you have any other comments to make?

### Measures

We chose to combine three questionnaires to gain more detailed insights from patients. We first used the system usability scale (SUS) questionnaire (55) to measure the usability of the conversational agent. SUS is a highly robust and versatile tool for usability professionals. The usability questions are composed of five attributes: learnability, efficiency, memorability, errors, and satisfaction. The questionnaire consists of 10 questions on a 5-point Likert scale. High scores (min. 0, max. 4) indicate high usability of the chatbot. A total SUS score above a 68 is considered above average, and anything below 68 is considered below average (56).

Then, to assess patients' perceived usefulness of the chatbot, consistently with the concept of patient empowerment, we used the Usefulness Scale for Patient Information Material (USE) questionnaire (57). Usefulness is measured on a global scale over three subscales which assess cognitive, emotional and behavioral subdimensions. The USE questions consist of 9 questions on a 5-point Likert scale. High scores (min. 0, max. 4) indicate high usefulness of the chatbot. Total maximal score is 36.

### Prototype Design

The high-fidelity prototype of TREVOR was developed with Chatfuel (58), the leading chatbot development platform for Facebook Messenger (59). This platform allows to design fully automated and script-based conversational agents. We chose to develop for Facebook Messenger because it is the second most downloaded messaging app after WhatsApp (47), and development for the latter was not yet available at the time.

TREVOR intends to support the daily lives of people with sickle cell disease. Its main goal is to help them better understand how to avoid triggering vaso-occlusive crises. To achieve this, the robot first asks patients to enter a comprehensive set of information including socio-demographics (i.e., educational attainment, location), prescribed treatments, physical and emotional health status, measurable biomarkers (e.g., oxygen saturation, basal hemoglobin level), history of complications, role functioning, self-care practices, empowerment levels, and exposure to potential triggers of VOCS (e.g., physical activity, sleep quality, dietary habits, exposure to cold). Then, the chatbot propose patients to:

- Identify what are the best self-management practices within the patient community;
- Compare their own self-care practices against those of the community;
- Assess their level of empowerment and identify their weaknesses;
- Learn how to adopt patient-recommended self-care practices through educational modules;
- Receive therapeutic accompaniment by a healthcare provider or an expert patient;
- Join online peer-support patient communities.

Participants are also informed that the program should not be used as a replacement for standard care and are urged to make an emergency call or contact their dedicated healthcare provider if necessary.

**Figure 1:**
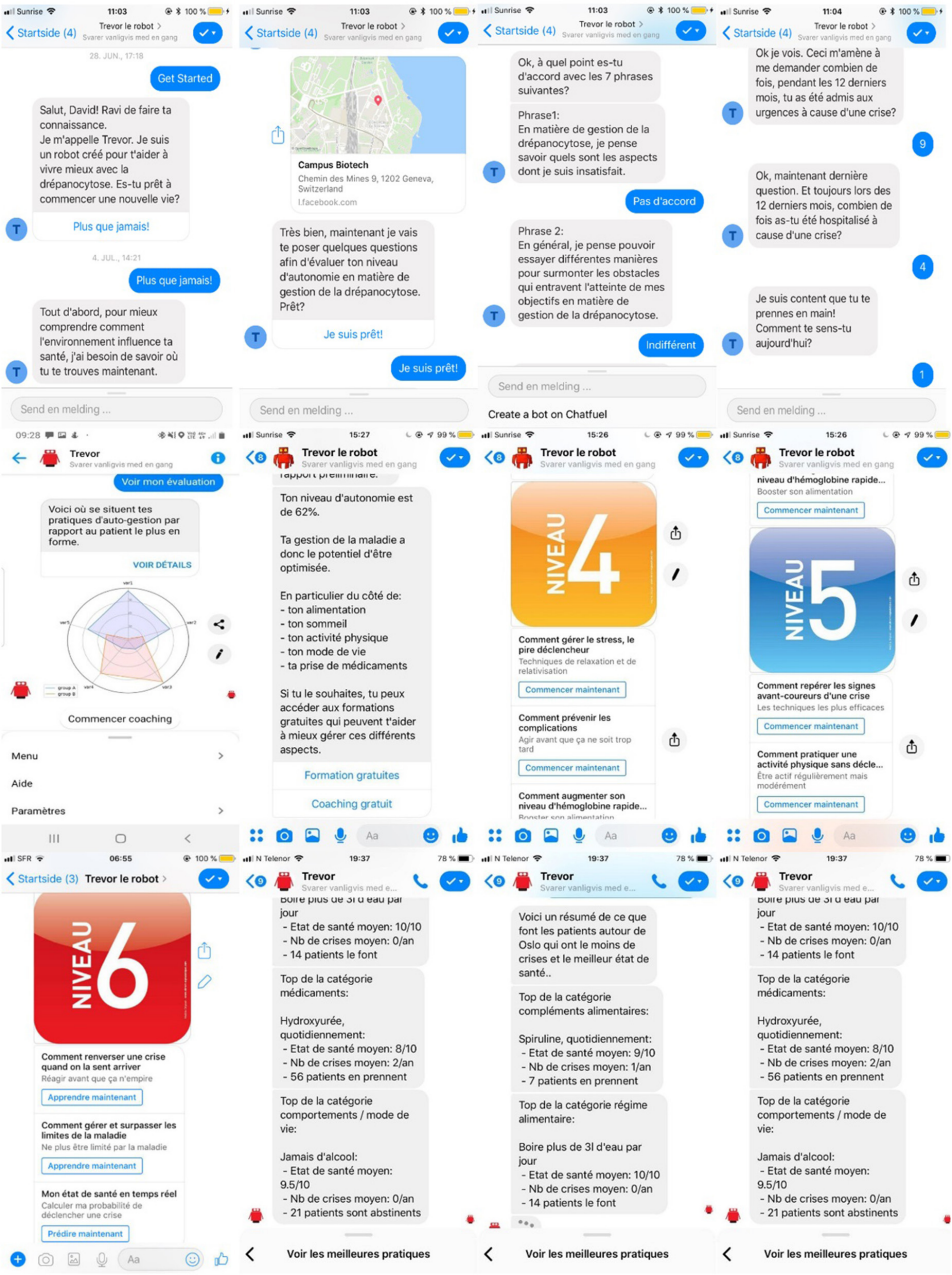
Snapshots of TREVOR conversational agent.

To support establishment of an emotional bond, enhance user engagement, and motivate patients, TREVOR addresses participants' accountability by referring to earlier data entered, tasks or activities performed (e.g., “Hi Robert, would you like to share more about your dietary habits?”). Furthermore, each message sequence begins with a warm greeting, in which the chatbot enquires about the participant's mood and replies in an empathic way (e.g., “Hello Alexandra, are you feeling better since last time we talked?”). Dialogues have been designed using the World Health Organization' handbooks on how to implement text-based mHealth interventions (60–65). In addition to text messages, media (i.e., videos clips, hyperlinks, audio messages) can be provided to support content delivery. Figure 1 provides representative snapshots of conversational interactions with TREVOR.

### Analysis

Descriptive statistics were computed for demographic and the survey data using STATA version 15 (StataCorporation, College Station, TX, USA). Data are presented as mean (SD) or number (percentage). Transcripts were organized and coded using ATLAS.ti version 8.3.20.0 (ATLAS.ti Scientific Software Development GmBH, Berlin, Germany). An inductive thematic analysis was applied to the data, and a coding framework was developed iteratively during analysis using the guidelines and checklist from Braun et al. (66). Once coding was complete, key themes were identified, explored, and interpreted by DZI. Emerging patterns were clustered together and checked for variability and consistency. Themes were interpreted by reading the codes back and forth.

## Results

### Participant Characteristics

Thirty-three patients participated in the test and two-third were females. Median age of the participants was 38 years. In addition, the majority of participants (23/33, 70%) were active, either studying or employed. More than half of patients (21/33, 64%) were affected by the most clinically severe SCD genotypes (Hb SS, HbSβ0). See Table 3 for further details.

**Table 3:** Characteristics of the study participants.

**Characteristic**	**Value (*N* = 33)**
**Gender**, ***n*** **(%)**	
Females	22 (66.67)
Males	11 (33.33)
**Age, years, mean (SD; range)**	37.37 (11.98; 19–59)
**Genotype**, ***n*** **(%)**	
Hb SS	19 (57.58)
Hb SC	11 (33.33)
HbSβ0	2 (6.06)
Hb S Lepore	1 (3.03)
**Children in household**, ***n*** **(%)**	
Yes	12 (36.36)
**Employment status**	
Student	30.3
(Self) Employed	39.4
Unable to work	12.1
Unemployed	9.1
Homekeeper	9.1
**Country of residence**, ***n*** **(%)**	
Guadeloupe	28 (84.84)
Martinique	5 (15.16)
**Smartphone Operating System**, ***n*** **(%)**	
Google Android	30 (90.9)
Apple iOS	3 (9.1)
**Years since using current Smartphone**, ***n*** **(%)**	
Less than 1 year	3 (9)
1 to 2 years	2 (6)
More than 2 years	28 (85)
**Frequency of Smartphone usage**	
Often (daily)	29 (88)
Regularly (several times a week)	2 (6)
Sometimes (1 to several times a month)	2 (6)
**Preferred messaging app**	
WhatsApp	26 (79)
Facebook Messenger	2 (6)
E-Mail	4 (12)
No preference	1 (3)
**User of existing mHealth app for SCD**	
SickleOScope	2 (6.06)
DrepaCare	4 (12.12)

### Perceived Usability and Usefulness

The results of the usability questionnaire are summarized in Table 4. Only two patients (6.1%) gave a total score below average (67), respectively 55 and 65. Mean scores of each positive question were at least 3.2, which was above the midpoint of the scale 0 to 4. The maximum mean score of negative questions was 0.9, which was below the midpoint of the scale 0 to 4, and indicates little need for assistance. The average SUS score was 83 (SD 11) out of the total of 100, and the median was 85, indicating a very usable system. Among the 15 participants older than 40 years old, 7 (47%) gave a SUS score above the median, while among the 18 patients younger than 40 years old, 11 (61%) gave a SUS score above the median.

**Table 4:** Results of the SUS questionnaire.

**Question**	**Mean (SD)**
I thought the system was easy to use.	3.5 (0.7)
I would imagine that most people would learn to use this system very quickly.	3.5 (0.5)
I think that I would like to use this system frequently.	3.3 (0.5)
I found the various functions in this system were well integrated.	3.2 (0.7)
I felt very confident using the system.	3.2 (1.1)
I needed to learn a lot of things before I could get going with this system.	0.9 (1)
I think that I would need the support of a technical person to be able to use this system.	0.7 (1)
I found the system unnecessarily complex.	0.7 (1)
I thought there was too much inconsistency in this system.	0.7 (0.9)
I found the system very cumbersome to use.	0.6 (1)

“*Concrete questions and indications; Easy and intuitive” Patient 20, 32 years old*

“*Fast processing, accurate and consistent information. No complexity due to reading or using the application.” Patient 8, 19 years old*

The results of the usefulness questionnaire are summarized in Table 5. Only four patients (12%) did not find the information provided useful, giving a score below average of respectively 13, 14, 15 and 16 out of 36. The minimum mean score was 2.2, and the maximum mean score was 3.1. The average USE score was 25 (SD 6.4) out of the total of 36, and the median was 26, which shows that patients found the information provided by the system useful. Among the 15 participants who were older than 40 years old, 5 (33%) gave a usefulness score higher than the median. In contrast, among the 18 patients younger than 40 years old, 10 (56%) gave a score higher than the median.

**Table 5:** Results of the USE questionnaire.

**The chatbot…**.	**Mean (SD)**
…contains information I need.	3.2 (0.8)
…encouraged me to become more active in order to improve my condition.	3.1 (1)
…showed me how I can contribute to the success of the treatment.	3.0 (0.8)
…has given me courage.	2.9 (0.9)
…helps me to participate in decisions made about my treatment.	2.8 (1)th
…helped me to understand the treatment options.	2.7 (1.2)
…has given me the hope that I will feel better again.	2.7 (0.8)
…reduced my worries about my disease/illness.	2.4 (1.3)
…helped me to understand the disease/illness.	2.3 (1.2)

“*Rich information (with scientific sources)” Patient 20, 32 years old*

Among the 4 participants (12%) who did not find the information provided very useful, 2 shared more insights about what they would like to receive. One of these patients, aged 41, asked for more content related to ulcer treatment. The other patient, aged 37, asked to receive more information about dietary supplements and phytomedicines for SCD. Finally, several patients (6/33, 18%) particularly appreciated the empathy conveyed by the chatbot:

“*It looks like we are communicating with someone who understands our health status” Patient 23, 37 years old*

### Debriefing Survey and Suggestion for Improvements

Twenty-four patients (73%) commented positively on the convenience of the design (e.g., ease of use, fun). Twenty-seven patients (82%) found the content particularly useful or interesting. Table 6 lists patients' suggestions for improvements in seven categories, for items that were expressed by more than one participant. Regarding content, more than one patient suggested to receive information about how to prevent and early detect VOCs, learn more about research advances, or learn more about how to manage their physical limitations (e.g., while hiking). Table 7 lists the suggestions for novel features extracted from the answers to open-ended questions. Regarding usability, the most typical request (10/33, 30%) for design improvements clustered around more flexibility in the choice of answers:

**Table 6:** Summary of suggestions of improvements.

**Suggestion**	**Number of quotes**
Enhance diversity of questions and choices of answers	6
Ease the modification of answers	4
Receive additional content	4
Improve readability / esthetics	3
Develop a standalone application	3
Have casual conversations with the chatbot	2

**Table 7:** Patients' suggestions for information that TREVOR should provide.

**Suggestion**	**Number of quotes**
How to prevent VOCs?	3
How to stop a VOC?	3
What are new research advances?	3
At what level are my important biomarkers (e.g., oxygen saturation, hemoglobin)?	2
Can I do a mountain hike /physical exercise today?	2
What are the new / best treatments?	2
What should I eat / drink?	2
How to early detect VOCs?	1
How to erase taboos and stigma?	1
What is my life expectancy?	1

“*Sometimes there are several response options but you can only choose one” Patient 31, 19 years old*

Some participants (3/33, 9.1%) felt that the readability of the context displayed was not optimal:

“*It might be preferable to create a standalone app, for better aesthetics” Patient 1, 36 years old*

Furthermore, some respondents (4/33, 12%) proposed to add more content:

“*Give advices to avoid reckless behaviors and inform about what to do when I have been reckless.” Patient 26, 46 years old*

From a usability point of view, some respondents (4/33, 12%) wished to be able to modify their answers more easily:

“*For corrections in case of input error, just go back straight to the error” Patient 30, 37 years old*

Although there exist some design issues, these problems did not cause severe inconveniences in using the chatbot. Both quantitative and qualitative results reflected that participants had a high intention to use TREVOR once ready:

“*Yes, I will use this app regularly, it contains information that are useful for me daily” Patient 4, 26 years old*

Overall, participants were satisfied by the chatbot, appreciated its ease of use and the content offered:

“*I like it a lot, it is very practical, interactive, fun, and easy to use” Patient 2, 59 years old*

Patients were particularly enthusiastic to be able to converse with the system anonymously:

“*As a strong point, it is really the anonymity” Patient 10, 29 years old*

### Unexpected Observations

Four patients (12%) encountered a bug while performing the tasks. The chatbot would not respond anymore until they closed and re-opened the conversation. This issue only appeared with Huawei smartphones. Two patients (6.1%) were assisted by their caregiver because of sight issues. Finally, one patient (3%) reported difficulties in understanding the meaning of some questions.

## Discussion

### Principal Findings

A small convenience sample of adults and young adults with SCD was recruited to examine the usability and usefulness of TREVOR, a conversational agent we designed to support SCD self-management. We used mixed-methods, combining quantitative and qualitative data. Following the evaluation, quantitative results showed high usability and usefulness scores. Qualitative findings provided insights into usability issues, usefulness and suggestions of improvements and new features. This study adds to the currently limited body of knowledge of chatbots for chronic disease health coaching, and suggests that conversational agents are welcomed by AYA with SCD.

As it is often the case in SCD research (68), women were predominant among participants, and men were under-represented (67). This suggests more incentivization (e.g., build a personal rapport, offer compensation) to male individuals with SCD may be necessary (69).

Consistently with most smartphone users (47), the large majority of respondents (88%) were daily users of messaging apps, and particularly WhatsApp. Although our prototype was available only on Facebook Messenger, patients did not suffer from an adaption period and felt confident using it. This is likely due to the similarity of conversational interfaces. In addition, most participants imagined that most other patients would learn to use the system very quickly. Congruently with prior research showing that chatbots are highly usable due to their simple and familiar user interface (70), our findings suggest that chatbots for chronic disease self-management can have high acceptance rates and usability scores.

An important finding is that the highest USE score was about the question: “the chatbot contains information I need.” This indicates that patients value learning from other patients' experiential knowledge, and in particular learning about what could be effective self-care practices. As well, participants seemed to appreciate receiving evidence-based knowledge on recommended self-management. As with other patient communities such as PatientsLikeMe or Crohnology (71), such features could inspire patients to participate in and contribute to the growth of commons-based peer production models and community-based research (72). However, some participants (12%) did not find the information provided particularly useful. Due to their age and according to what some suggested, it is likely that they already had good disease-specific knowledge, and therefore simply wished to have more specific information.

Participants emphasized having been able to learn about their disease in a ludic and empathetic way. This finding is not surprising since TREVOR has been conceived to have an empathetic personality, and since personification is known to lead to high user satisfaction, user engagement and dialogue quality (73). In addition, since motivation is an important factor for patient empowerment, can exert an influence on self-management, and on the adoption of healthy behaviors (74), such personalization may be able to enhance patient engagement in the long-run (75). Consequently, it is important to further investigate similar approaches to better understand how using chatbots for mHealth interventions could have a positive impact on health outcomes.

Patients highlighted their desire to prevent and detect early VOCs. Currently, a growing number of mHealth apps are being developed, but none provides information on such a patient-important aspect of disease management. We think that since our findings indicate that conversational agents could be a useful channel of communication for mHealth interventions, further research should focus on how to provide targeted information on particular domains of self-care management such as the prevention of VOCs.

By implementing such features, future chatbots could increase their utility. Therefore, in our opinion, it is particularly important to conduct profound research, both in low- and middle- as well as in high income countries, to collect information about the various ways that patients self-manage all over the world. Afterwards, giving back such information through a conversational agent could be effective.

## Limitations

We acknowledge that it is difficult to generalize our findings because of small sample size and non-probability sampling methods. There is likely a selection bias. Since some of the participants were recruited by contacting patient associations, were asked to bring their own smartphone, and given most were students or employed (presumably people with good health functioning and self-efficacy), it may have attracted the most active and motivated ones, and those particularly at ease with social media apps. However, in view of the results, participants may be willing to inform other patients about the potential benefits of TREVOR, helping to recruit more diverse participants in future studies. Precise interpretation remains difficult since research applying a socio-demographic determinants of health lens in people with SCD in French West Indies is lacking. Highly acceptable digital health tools such as TREVOR could be useful to collect real-life data and socio-demographic data in such studies.

Another limitation is that patients did not use the app for a prolonged period of time. We think it would also have been useful to determine the average chatbot session length. Therefore, results should be considered with due caution.

The principal investigator being an expert patient, it is likely that it enhanced participants' interested, and that patients were particularly open-minded in contributing to the development of new means of health service delivery designed to meet their needs.

Our study is limited by the participant characteristics, which may not reflect the whole population of adults with SCD. First, the study was carried out only on a West Indian SCD population, but this should have little impact given the similarity of the challenges faced by adults with SCD all over the world (76).

It is also important to note that conversations have been designed by French-speaking people from a French cultural influence. Adaptations to make conversations appropriate to different languages and culture could be needed in future versions. Another limitation is that the reading level was not assessed. However, the text was reviewed by nurses practicing patient therapeutic education. Then, given participants' relatively young median age (38), it is likely that patients were already comfortable using messaging apps.

Although the sample size is small, patients above the median age found the system slightly less easy to use and less useful, suggesting that chatbots may be less adapted to older populations. Furthermore, since our sample did not include younger people with SCD, and since the chatbot was conceived for adults and young adults, we do not know if pediatric patients or their caregivers would have preferred other communication channels or different content.

In order to facilitate user experience, we did not enable the possibility for patients to enter free-text. However, some patients wished to have this possibility. Finally, extensive tests with various smartphone brands will be needed in order to comprehensively debug the system.

### Recommendations for Future Design and Implementation Research

On the basis of the findings of this study and patients' suggestions of improvements, we formulate eight recommendations for the future developments of chatbots:

1. Simplify the readability of the system (77):
  - Patients reported that displaying a long list of information could be challenging to read. We recommend developers to make sure that content is written at an appropriate reading level. As well, since cognitive function may be decreased in symptomatic people with SCD (78, 79), making the content more readable could be beneficial to patients. This could be done for instance by providing more media (e.g., images, infographics, animations), short and simple sentences, by enabling text-to-speech features, by making sure the user has enough time to read the text and respond to the message, and by developing content in collaboration with patients with various reading skills, health literacy levels and neuropsychological problems.
2. Improve the flexibility and efficiency of user input:
  - combine text-based interface with buttons and media;
  - add auto-suggestion buttons (e.g., using Natural Language Processing);
  - enable multiple-choice questions;
  - enable casual conversations and free-text answers.
  - enable modification of previously entered answers and explicitly design dialog failures interactions (e.g., automatically go back to the point of failure);
  - integrate automatic data collection of important biomarkers (e.g., oxygen saturation) from wearables and health-tracking platforms (e.g., Google Fit, Validic) (80, 81).
3. Address various levels of patient health literacy (82):
  - One patient (3%) had troubles interpreting the meaning of some questions. Prior research has demonstrated that people with SCD had suboptimal levels of health literacy (83). Therefore, it is important to tailor health information to the skills of patients. As McClure et al. (84) demonstrated, several tools can be used to assess patient education material. As well, Alberts et al. (85) successfully used the Newest Vital Signal instrument to measure health literacy and numeracy in patients with SCD (86). We recommend further studies to pay attention into matching the language and content with patient abilities, and to provide links to additional explanations (87).
4. Encourage participatory research and co-design:
  - To satisfy highly literate patients and help improve the quality of the content provided, we recommend researchers to invite such knowledgeable patients into the elaboration of mHealth chatbots.
5. Disseminate chatbots on as many messaging platform as possible, including as a native app:
  - To provide patients with an agreeable user experience on their favorite messaging app, attention should be paid on measuring, and comparing performance on various messaging platforms. This could be achieved by comparing metrics such as usability, esthetics, speed, or by investigating alternative methods for conversational user interface evaluation (88, 89).
6. Build a knowledge base into which the chatbot could tap:
  - Since most of the suggestions of new features were related to answering specific questions about SCD, building such a base could contribute to the evidence base.
7. Provide effective chatbot discovery:
  - Chatbots are not listed in Google Play or Apple App Stores. Thus, since chatbot directories are manifold (90), a version of the chatbot could be developed as a native app for Android or iPhone users to facilitate discovery.
8. Add empathetic small-talk and psycho-social support capabilities;
  - Since some patients have asked to be able to discuss about life matters, future chatbots should include the possibility to answer to a variety of conversation types, or redirect the conversation to humans (91).

## Conclusion

Self-management of people with SCD is a never-ending task. It is therefore important to seek to develop systems that can fit into patients' daily life with the least disturbances possible. Messaging apps are among the most popular smartphone apps. Therefore, building mHealth interventions that meet patients directly where they are may facilitate adoption and long-term engagement.

This study is the first to contribute to the evidence base regarding the utility and effectiveness of chatbots for adults with SCD. As well, findings contribute to the growing literature demonstrating how usability assessment of mHealth apps provides invaluable information for iterative developments. This study was achieved by testing a high-fidelity prototype of a mHealth coaching app in terms of usability and usefulness if information provided. We used a non-clinical convenience sample of adults with SCD living in the French West Indies.

Quantitative results suggest that chatbots for health coaching can be easy and fun to use, while providing useful support for patient empowerment. In the qualitative phase, participants expressed their enthusiasm using the system, and emphasized on the usefulness of such system for disease-specific knowledge acquisition. The large majority of participants found the content interesting and useful to learn more about recommended self-care practices related to the prevention of symptoms.

However, to better understand if chatbots for SCD can become an useful complement to clinical care, controlled studies will be needed to evaluate over longer periods aspects such as clinical utility, clinical safety, acceptability, usage, or engagement. Finally, future studies could attempt to recruit participants from a wider range of backgrounds, and other in-depth evaluation methods could be carried out.

## Data Availability Statement

The original contributions presented in the study are included in the article/Supplementary Material, further inquiries can be directed to the corresponding author/s.

## Ethics Statement

Ethical review and approval was not required for the study on human participants in accordance with the local legislation and institutional requirements. The patients/participants provided their written informed consent to participate in this study.

## Author Contributions

DZI is the principal investigator and made a direct and substantial contribution to this work by providing the project idea, study topic, conception, and design of the study and also conceived the prototype and contributed to data collection and analysis. MDHD and MR significantly contributed to patient recruitment and the usability tests. GH and CL supervised the study and provided critical revisions that are important for the intellectual content of the manuscript. MH-D, MR, GH, and CL revised the manuscripts. All the authors have read and approved the final version of the manuscript.

## Conflict of Interest

The authors declare that the research was conducted in the absence of any commercial or financial relationships that could be construed as a potential conflict of interest.
